# TRIM54 alleviates inflammation and apoptosis by stabilizing YOD1 in rat tendon-derived stem cells

**DOI:** 10.1016/j.jbc.2023.105510

**Published:** 2023-11-30

**Authors:** Hua Chen, Xiaofeng Chen, Ling Yang, Shiyang Sheng, Jianshe Yang, Yong Lu, Yangbai Sun, Xiaoping Zhang, Chaoyin Jiang

**Affiliations:** 1 Department of Orthopedics, Shanghai Sixth People's Hospital Affiliated to Shanghai Jiao Tong University School of Medicine, Shanghai, China; 2 Department of Orthopaedic Surgery, Hainan Province Clinical Medical Center, Sanya, China; 3 Haikou Orthopedic and Diabetes Hospital of Shanghai Sixth People's Hospital, Haikou, China; 4 Department of Nuclear Medicine, Shanghai Tenth People's Hospital, Tongji University, Shanghai, China; 5 Department of Radiology, Ruijin Hospital, School of Medicine, Shanghai Jiao Tong University, Shanghai, China; 6 Department of Radiology, Ruijin Hospital Luwan Branch, School of Medicine, Shanghai Jiao Tong University, Shanghai, China; 7 Department of Musculoskeletal Oncology, Fudan University Shanghai Cancer Center, Shanghai, China; 8 Department of Oncology, Shanghai Medical College, Fudan University, Shanghai, China; 9 The Institute of Intervention Vessel, Tongji University School of Medicine, Shanghai, China

**Keywords:** TRIM54, YOD1, tendinopathy, tendon-derived stem cells, apoptosis

## Abstract

Tendinopathy is a disorder of musculoskeletal system that primarily affects athletes and the elderly. Current treatment options are generally comprised of various exercise and loading programs, therapeutic modalities, and surgical interventions and are limited to pain management. This study is to understand the role of TRIM54 (tripartite motif containing 54) in tendonitis through *in vitro* modeling with tendon-derived stem cells (TDSCs) and *in vivo* using rat tendon injury model. Initially, we observed that TRIM54 overexpression in TDSCs model increased stemness and decreased apoptosis. Additionally, it rescued cells from tumor necrosis factor α–induced inflammation, migration, and tenogenic differentiation. Further, through immunoprecipitation studies, we identified that TRIM54 regulates inflammation in TDSCs by binding to and ubiquitinating YOD1. Further, overexpression of TRIM54 improved the histopathological score of tendon injury as well as the failure load, stiffness, and young modulus *in vivo*. These results indicated that TRIM54 played a critical role in reducing the effects of tendon injury. Consequently, these results shed light on potential therapeutic alternatives for treating tendinopathy.

Tendinopathy is characterized by tendon injuries caused by overuse which leads to athletes and the elderly chronic pain (1). The etiology of tendon injuries is still subject to debate. Initially, it was believed that inflammation was the primary cause of tendinitis; However, as research has progressed, there is a growing consensus that inflammation and degeneration together contribute to the prognosis (2). Tendons consist of collagen fibril bundles that become encased in endotenon and then epitenon. Overuse and repeated stretching of tendons triggers the release of pro-inflammatory factors, which induce metalloproteinases and result in collagen degradation. Therefore, with prolonged use, degeneration accumulates, leading to tendinopathy (3).

Tripartite motif (TRIM) proteins play crucial roles in numerous essential biological processes, particularly as immune system components in regulating oncogenesis pathways (4). This family consists of approximately 80 E3 ligase proteins with diverse functions, including cell proliferation, migration, and differentiation (5). E3 ligase is one of the most prevalent ubiquitination enzymes that regulates numerous genes and processes (6). The TRIM protein family includes an N-terminal RING finger domain, box domains, and a coil region. The C-terminal region of proteins is involved in DNA and RNA binding, protein–protein interactions, and chromatin-mediated transcriptional regulation (7). Numerous TRIM members have been identified as playing crucial roles in the stemness of cells. TRIM6 plays a critical role by interacting with c-myc proto-oncogene and maintains the mouse embryonic stem cell pluripotency (8). TRIM28 inhibits cell differentiation by promoting the expression of Oct4, Nanog, and Sox2 (9). Moreover, TRIM19 is highly expressed in hematopoietic stem cells, and its absence reduces their survival significantly (10). However, relatively less is known about TRIM54's function in stem cell research.

In addition to being regulators of numerous molecular pathways, TRIM proteins are implicated in the development of complex disease mechanisms. TRIM11 is known to activate STAT3 and the vascular endothelial growth factor pathway, and its overexpression has been associated with lung and hepatocellular carcinoma (11). Similarly, TRIM54 has been found to activate wingless-related integration site β-catenin *via* Axin1 turnover, thereby promoting the proliferation of hepatocellular carcinoma cell lines (12). In addition, numerous TRIMs have been identified as important regulators of inflammation (13). Interestingly, some TRIMs are pro-inflammatory, whereas others are anti-inflammatory. By activating apoptosis and the nuclear factor kappa-light-chain-enhancer of activated B cells (NF-κB) pathway, TRIM8 positively regulates cerebral ischemia reperfusion injury (14). However, TRIM72 significantly attenuates neurotoxicity, inflammation, and apoptosis by inhibiting NF-κB phosphorylation (15). In this study, we identified that TRIM54 upregulates proliferation, maintains stemness, and decreases inflammation in tendon-derived stem cells (TDSCs). Additionally, we confirmed that TRIM54 significantly reverses inflammation and injury using *in vivo* tendonitis models. Consequently, this study contributes to the comprehension of TRIM54's role in tendinopathy and identifies a novel treatment axis for tendon injury.

## Results

### TRIM54 levels were significantly downregulated in human tendinopathy samples

**Figure 1:**
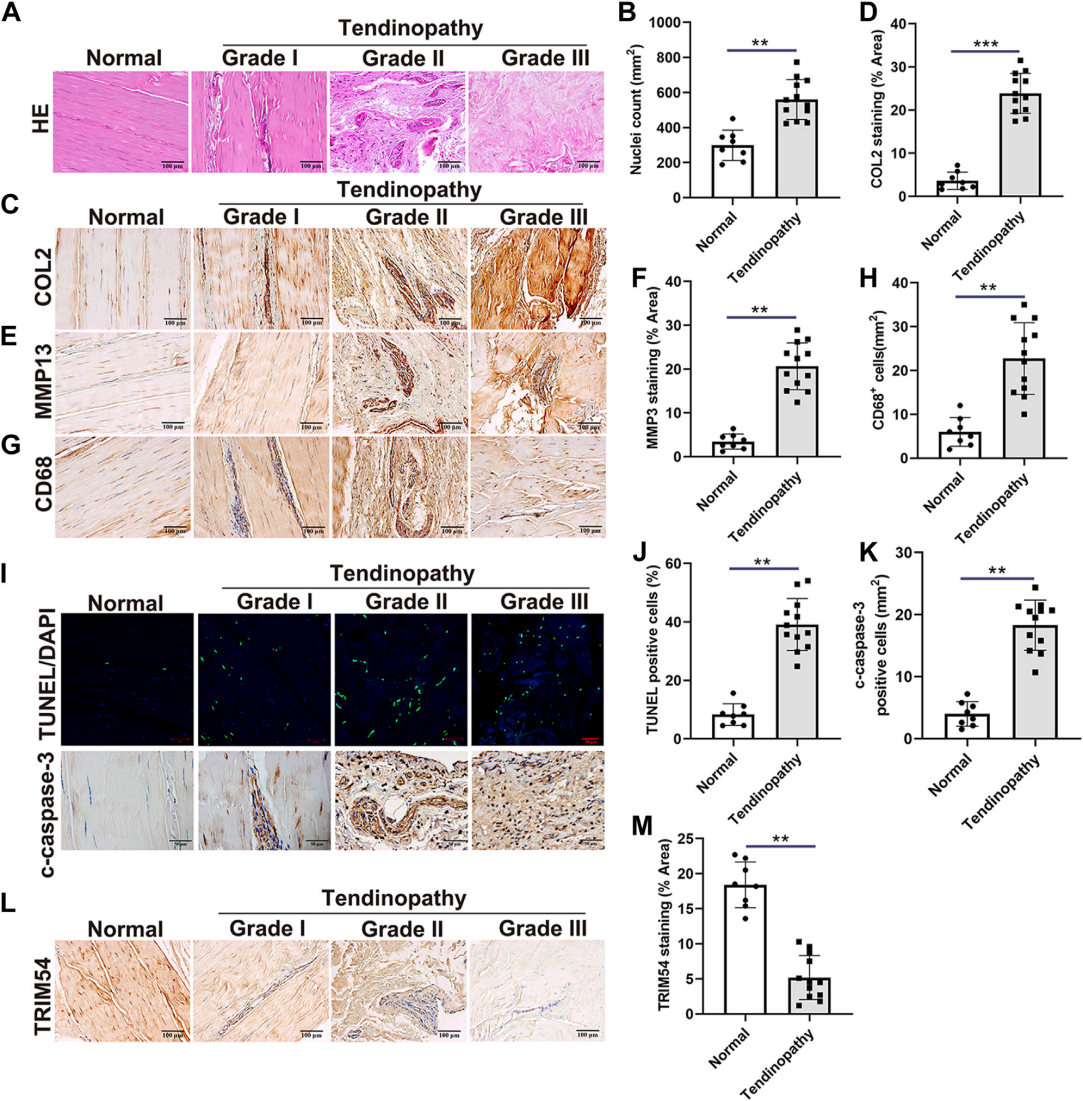
**TRIM54 levels are significantly decreased in human tendinopathy samples.***A* and *B*, HE staining of normal tendons and tendinopathic tendons (Grade I-III) along with quantification of nuclei per bone marrow area (mm^2^, Scale bar, 100 μm). *C* and *D*, immunohistochemical staining of COL2 in normal tendons and tendons with different grades of tendinopathy, as well as the staining area of each tissue area of COL2 (mm^2^, Scale bar, 100 μm). *E* and *F*, immunohistochemical staining of MMP13 in normal tendons and tendons with different grades of tendinopathy, as well as the staining area of each tissue area of MMP13 (mm^2^, Scale bar, 100 μm). *G* and *H*, immunohistochemical staining of CD68 in normal tendons and tendons with different grades of tendinopathy, CD68^+^ macrophages (Scale bar, 100 μm). *I*–*K*, TUNEL staining and c-caspase-3 immunohistochemical staining in normal tendons and tendons with different grades of tendinopathy (Scale bar, 50 μm). *L* and *M*, immunohistochemical staining of TRIM54 in normal tendons and tendons with different grades of tendinopathy, as well as the staining area of each tissue area of TRIM54 (mm^2^, Scale bar, 100 μm). Normal tendons n = 8; tendinopathic tendons n = 12. Statistical significance was determined using unpaired Student’s *t* test. Data are presented as mean ± SD. ∗∗*p* < 0.01, ∗∗∗*p* < 0.001. HE, hematoxylin–eosin; MMP13, metalloproteinase 13; TRIM54, tripartite motif containing 54.

**Supporting Figure S1:**
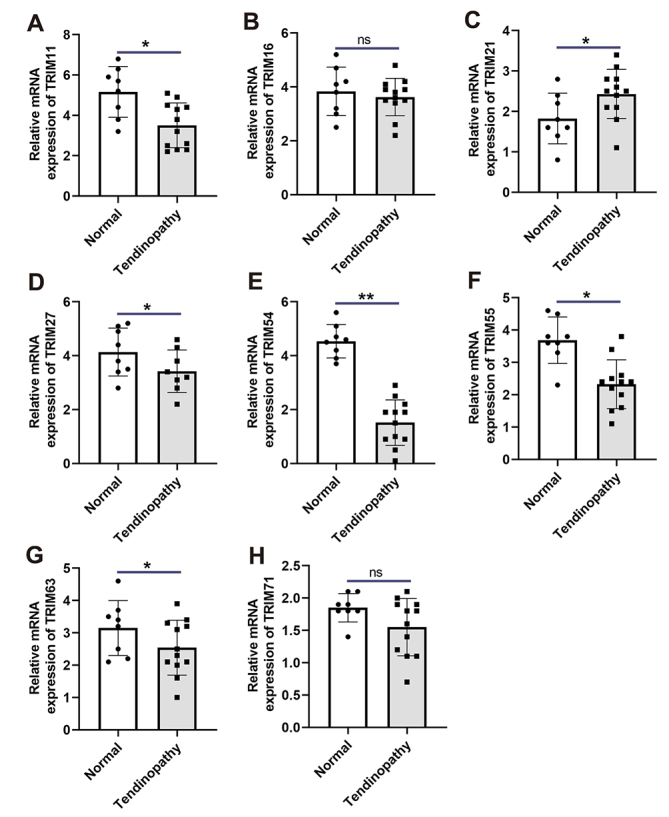
**The mRNA expressions of eight TRIM family members (TRIM11, TRIM16, TRIM21, TRIM27, TRIM54, TRIM55, TRIM63, and TRIM71) in normal tendon and tendinopathic tendon.***A*–*H*, the mRNA expressions of eight TRIM family members in normal tendons and tendons with different grades of tendinopathy were measured by qRT-PCR. Normal tendons n = 8; tendinopathic tendons n = 12. Statistical significance was determined using unpaired Student’s *t* test. Data are presented as mean ± SD. ∗*p* < 0.01, ∗∗*p* < 0.01 compared with the indicated groups. TRIM, tripartite motif.

Initially, we assessed and graded the tissue samples for tendinopathy (Fig. 1, *A* and *B*). This is supported by the immunohistochemical staining of collagen type 2, metalloproteinase 13, and CD68, which show increased staining in tendinopathic tendons compared to normal tendons (Fig. 1, *C*–*H*). Additionally, TUNEL staining and the immunohistochemical staining of cleaved-caspase-3 (c-caspase-3) suggest that there is increased apoptosis in tendinopathic tendons (Fig. 1, *I*–*K*). Furthermore, the qRT-PCR assays determined the mRNA expression levels of eight TRIM family members (TRIM11, TRIM16, TRIM21, TRIM27, TRIM54, TRIM55, TRIM63, and TRIM71) in normal tendons and tendinopathic tendons (Fig. S1). The results showed that TRIM11, TRIM27, TRIM54, TRIM55, and TRIM63 mRNA levels were considerably downregulated in tendinopathic tendons, while TRIM21 was upregulated. Further, TRIM54 was the one that changed the most substantially. Meanwhile, immunohistochemical staining of TRIM54 shows decreased staining in tendinopathic tendons compared to normal tendons (Fig. 1, *L* and *M*). These findings imply that TRIM54 may be involved in the development of tendinopathy and that more research into its function is needed.

### TDSCs maintain multipotent differentiation potential

**Figure 2:**
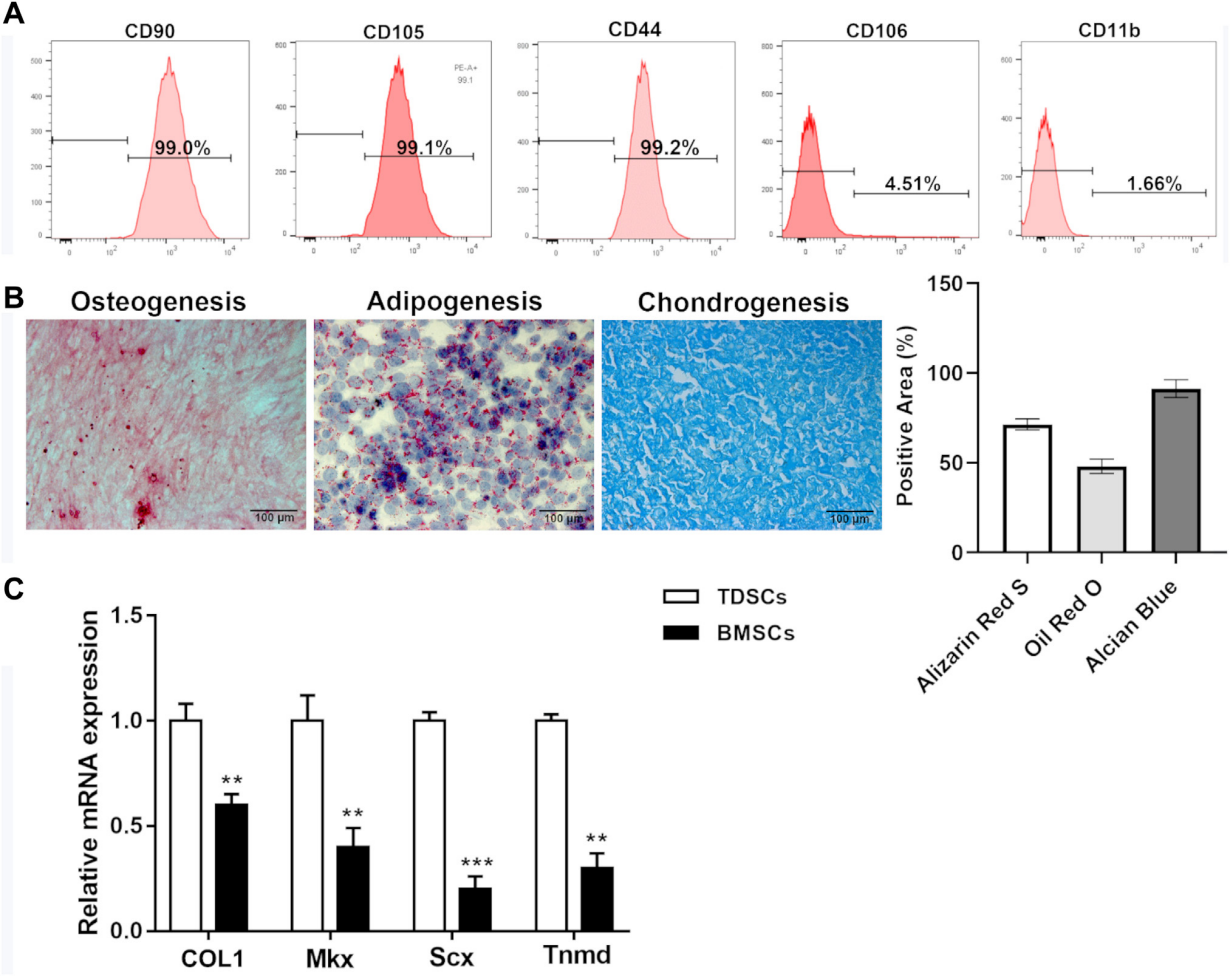
**Characterization of tendon-derived stem cells (TDSCs).***A*, representative flow cytometry data of TDSCs incubated with CD90, CD105, CD44, CD106, and CD11b (*red* for control and *blue* for fluorescent antibody). *B*, Alizarin Red S stain (osteogenesis), Oil Red O stain (adipogenesis), and Alcian Blue stain (chondrogenesis) (the scale bar represents 100 μm), and the quantification of the three stains. *C*, qRT-PCR of tenogenesis markers COL1, Mkx, Scx, and Tnmd. Differences between two groups were analyzed by unpaired Student *t* test. n = 3 per group. Data are presented as mean ± SD. ∗∗*p* < 0.01, ∗∗∗*p* < 0.001 compared with the indicated groups. COL1, collagen type 1; Mkx, Mohawk; Scx, scleraxis; Tnmd, tenomodulin.

To investigate the molecular cause of tendinitis, we extracted and characterized TDSCs from male Sprague-Dawley rats using flowcytometry. Initially, we observed that the cells were positive for stem cell precursor markers CD90, CD105, and CD44 while being negative for CD106 and CD11b (Fig. 2*A*). We next differentiated the cells into osteogenesis, adipogenesis, and chondrogenesis and stained them appropriately. Indeed, Alizarin Red S, Oil Red O, and Alcian Blue staining revealed that these cells could differentiate into all three cell types (Fig. 2*B*). Further, we completed the characterization of these cells by performing qRT-PCR and observed that cells expressed collagen type 1 (COL1), mohawk, scleraxis, and tenomodulin (Tnmd) (Fig. 2*C*).

### TRIM54 regulates proliferation, differentiation, and inflammatory responses in TDSCs

**Figure 3:**
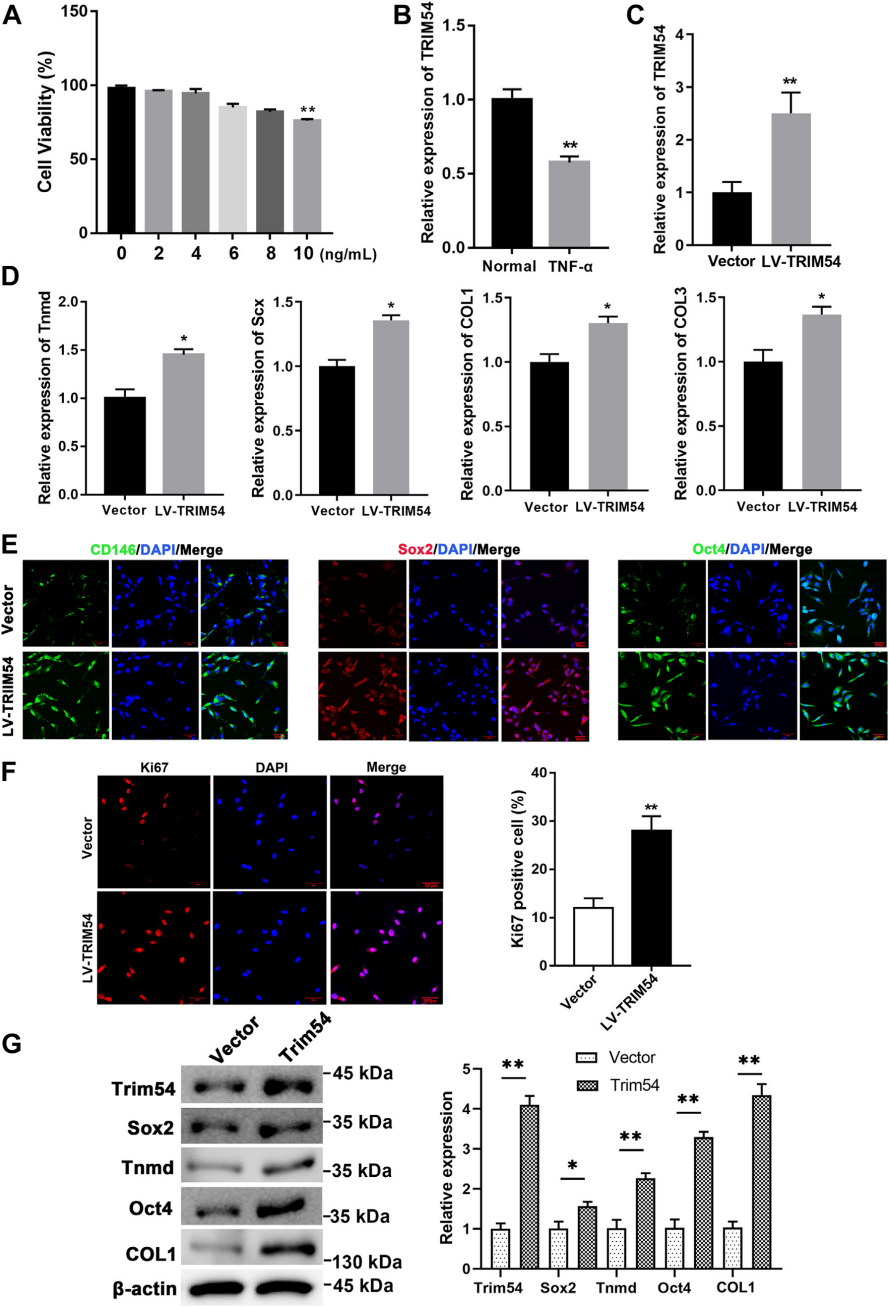
**TRIM54 regulates tenogenesis in TDSCs.***A*, TDSCs were incubated with various concentrations of TNF-α for 24 h, and CCK8 assay was set to measure the role of TNF-α on TDSCs viability. *B*, relative expression of TRIM54 after exposure to 10 ng/ml TNF-α. *C*, qRT-PCR detection of transfection efficiency after overexpressing TRIM54. *D*, qRT-PCR of tenogenesis markers (Tnmd, Scx, COL1, COL3). *E*, immunofluorescence staining of CD146, Sox2, and Oct4 in TDSCs overexpressing TRIM54 (Scale bar, 50 μm). *F*, immunofluorescence staining and quantification of Ki67 in TDSCs overexpressing TRIM54 (Scale bar, 50 μm). *G*, Western blotting and quantification analysis of TRIM54, Sox2, Tnmd, Oct4, and COL1 in cells overexpressing TRIM54 with respect to controls. n = 3 per group. Differences between two groups were analyzed by unpaired Student's *t* test. Data are presented as mean ± SD. ∗*p* < 0.01, ∗∗*p* < 0.01 compared with the indicated groups. COL1, collagen type 1; TDSC, tendon-derived stem cell; Tnmd, tenomodulin; TRIM54, tripartite motif containing 54.

**Supporting Figure S2:**
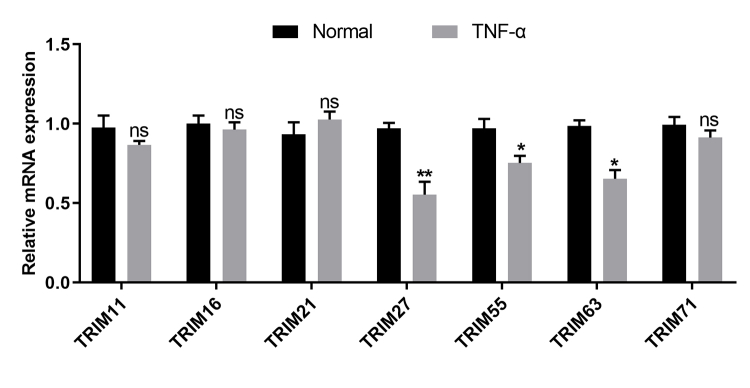
**Relative expression of TRIM family members (TRIM11, TRIM16, TRIM21, TRIM27, TRIM55, TRIM63, and TRIM71) after exposure to 10 ng/mL TNF-α. n = 3 per group.** Differences between two groups were analyzed by unpaired Student's *t* test. Data are presented as mean ± SD. ∗*p* < 0.01, ∗∗*p* < 0.01 compared with the indicated groups. TRIM, tripartite motif.

To model tendonitis, we induced inflammation in these TDSCs by exposing them to TNF-α. Initially, we incubated the cells with varying concentrations of TNF-α for 24 h and performed CCK8 assay to assess its effect on the proliferation of TDSCs. Indeed, the proliferation of TDSCs decreased as concentration of TNF-α increased (Fig. 3*A*). Further, we assessed the levels of TRIM54 in cells exposed to TNF-α and observed that cells expressed significantly low levels of TRIM54 in the presence of TNF-α (Fig. 3*B*). Similarly, we also assessed levels of other TRIM family members (TRIM11, TRIM16, TRIM21, TRIM27, TRIM55, TRIM63, and TRIM71) in the cells exposed to TNF-α (Fig. S2). To further elucidate its role on tendonitis, we overexpressed TRIM54 in TDSCs and confirmed its levels using qRT-PCR (Fig. 3*C*). We also observed that overexpression of TRIM54 significantly increased expression of tenogenesis markers such as COL1, collagen type 3, scleraxis, and Tnmd in TDSCs (Fig. 3*D*). Further, we also observed an increase in progenitor marker expression CD146, Sox2, and Oct4 in TDSCs overexpressing TRIM54 (Fig. 3*E*). Ki-67 staining of these cells indicated that overexpression of TRIM54 significantly increased proliferative capacity of TDSCs (Fig. 3*F*). Western blot results showed that overexpression of TRIM54 significantly increased the expression of Sox2, Tnmd, Oct4, and COL1 in TDSCs (Fig. 3*G*).

**Figure 4:**
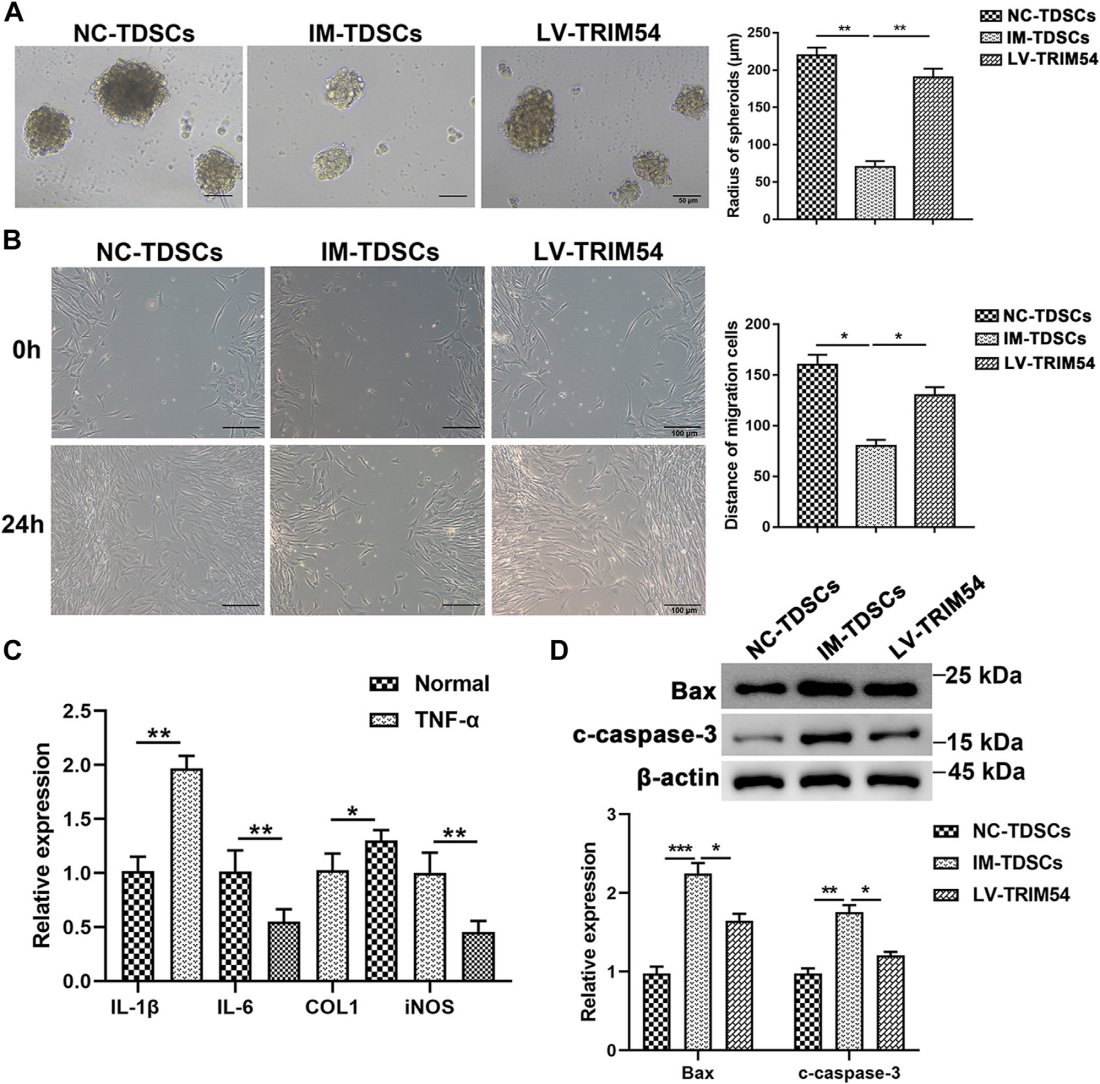
**TRIM54 overexpression downregulates TNF-α induced inflammation.***A*, sphere-forming assay and quantification analysis of NC-TDSCs, IM-TDSCs, and LV-TRIM54 TDSCs (Scale bar, 50 μm). Statistical significance was determined using one-way ANOVA and Tukey’s multiple comparisons test. *B*, wound scratch assay and quantification analysis of NC-TDSCs, IM-TDSCs, and LV-TRIM54 TDSCs (Scale bar, 100 μm). Statistical significance was determined using one-way ANOVA and Tukey’s multiple comparisons test. *C*, mRNA expression of inflammatory markers, IL-1β, IL-6, COL1, and iNOS were evaluated by using qRT-PCR. Differences between two groups were analyzed by unpaired Student's *t* test. *D*, Western blotting and quantification analysis of apoptosis markers, Bax and c-caspase-3, protein expression in TDSCs after exposure to TNF-α. Statistical significance was determined using one-way ANOVA and Tukey’s multiple comparisons test. n = 3 per group. Data are presented as mean ± SD. ∗*p* < 0.01, ∗∗*p* < 0.01, ∗∗∗*p* < 0.001 compared with the indicated groups. COL1, collagen type 1; IL, interleukin; iNOS, inducible nitric oxide synthase; TDSC, tendon-derived stem cell; TRIM54, tripartite motif containing 54.

We subsequently performed sphere formation assay and observed that TDSCs exposed to TNF-α (IM-TDSCs) formed significantly smaller spheres than normal TDSCs. However, when we overexpressed TRIM54 in TDSCs (LV-TRIM54), we observed formation of bigger spheres compared to IM-TDSCs (Fig. 4*A*). Wound healing assay also indicated that exposure to TNF-α significantly decreased the proliferation and migration of cells as shown by decreased wound closure. Subsequently, overexpression of TRIM54 in these cells significantly improved wound healing capacity (Fig. 4*B*). It was evident that exposure to TNF-α significantly increased interleukin (IL)-1β and COL1 levels, while decreasing IL-6 and inducible nitric oxide synthase levels (Fig. 4*C*). Further, we performed Western blotting analysis and observed that exposure to TNF-α significantly increased the expression of apoptotic markers Bax and c-caspase-3. Overexpression of TRIM54 clearly reversed these results wherein we observed a decrease in Bax and c-caspase-3 (Fig. 4*D*).

### TRIM54 interacts and ubiquitinates YOD1

**Figure 5:**
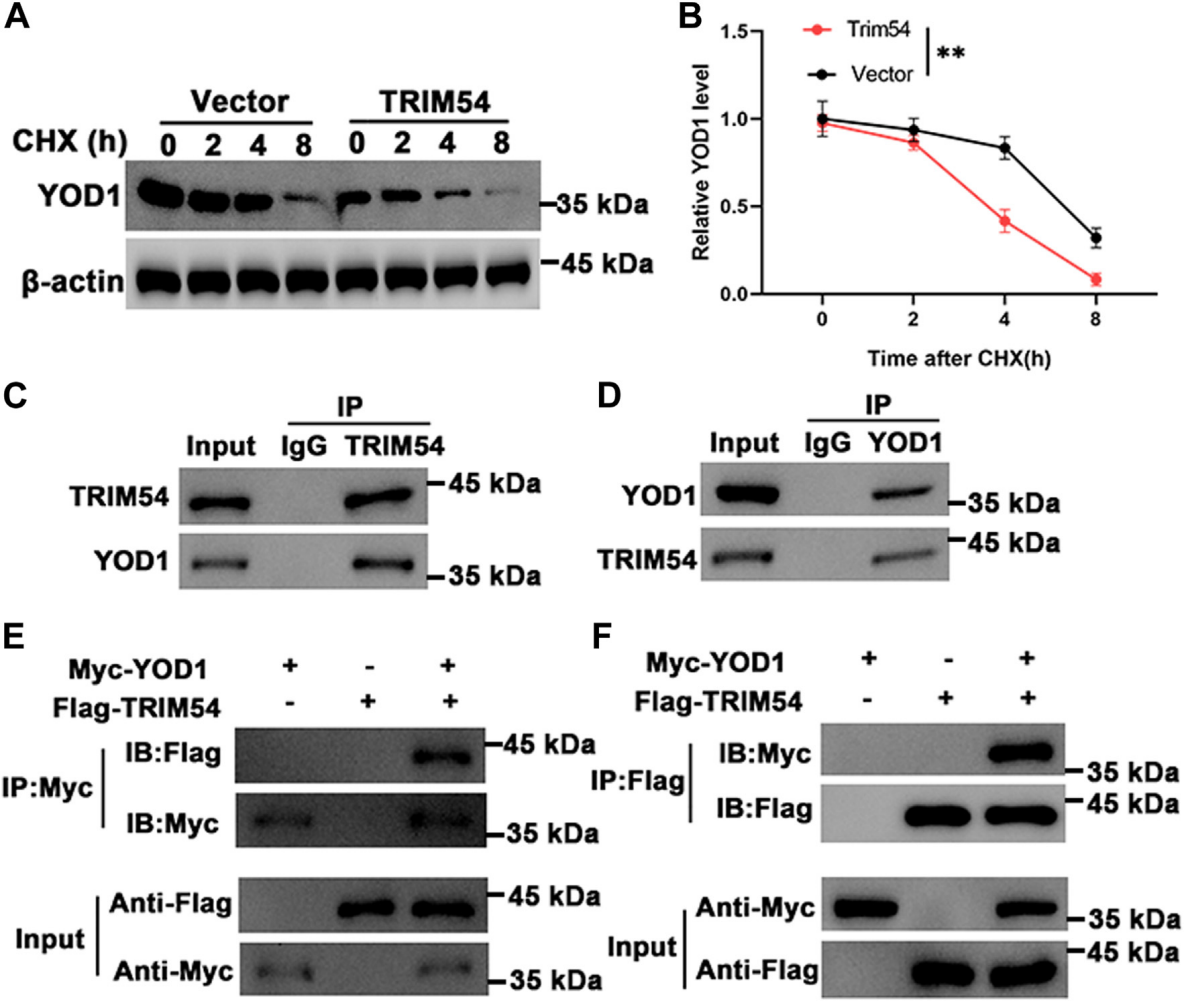
**TRIM54 binds and regulates YOD1 protein.***A* and *B*, detection of YOD1 protein levels in control and TRIM54 overexpressing TSPCs in the presence of CHX (10 μg/ml) for indicated time point. *C* and *D*, detection of endogenous protein interactions between TRIM54 and YOD1 in cells lysates. *E* and *F*, detection of exogenous protein interactions between TRIM54 and YOD1 in HEK 293T cells. n = 3 per group. Data are presented as mean ± SD. ∗∗*p* < 0.01 compared with the indicated groups. CHX, cycloheximide; TRIM54, tripartite motif containing 54.

**Figure 6:**
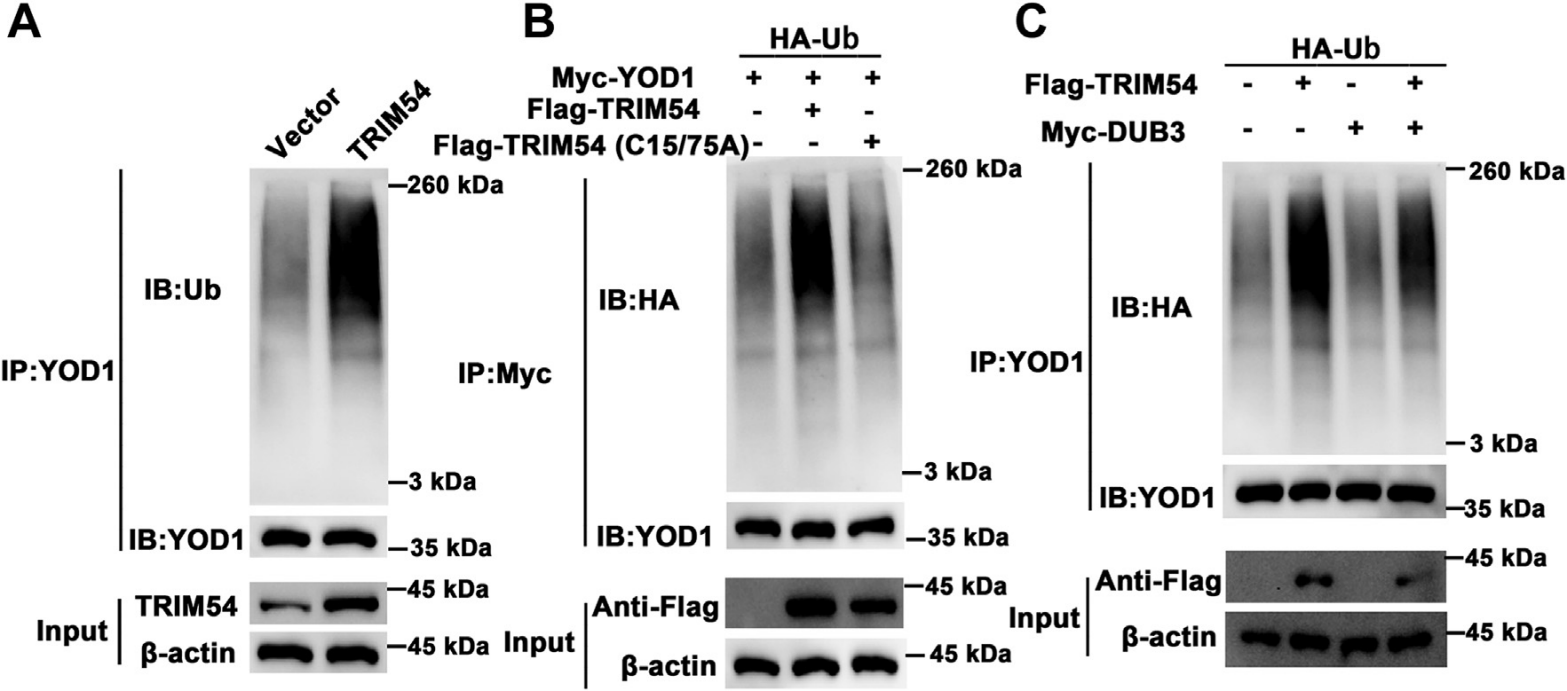
**TRIM54 regulates YOD1 through ubiquitination.***A*, evaluation of endogenous YOD1 ubiquitination in cells transfected with vector and LV-TRIM54. *B*, valuation of exogenous YOD1 ubiquitination in HEK 293T cells cotransfected with Flag-tagged TRIM54 (WT) or Flag-tagged TRIM54, HA-tagged Ub, and Myc-tagged YOD1. *C*, HEK 293T cells transfected with Flag-TRIM54, Myc-DUB3 as well as Ub, and then YOD1 ubiquitylation linkage was assessed. TRIM54, tripartite motif containing 54.

BioGRID Database Statistics predicted a possible interaction between TRIM54 and YOD1 (https://thebiogrid.org/), hence we wanted to assess YOD1’s role during inflammation. Initially, we used cycloheximide chase assay to illustrate the potential relation between TRIM54 and YOD1. We observed with increasing exposure to cycloheximide, YOD1 levels decreased significantly, which was even further decreased in cells overexpressing TRIM54 (Fig. 5, *A* and *B*). Further, we performed immunoprecipitation assay where protein immunoprecipitated using TRIM54, when assessed using Western blot showed the presence of YOD1 protein. Similarly, when protein was immunoprecipitated with YOD1 antibody and assessed using Western blot, we could clearly observe the presence of TRIM54 (Fig. 5, *C* and *D*). We next performed co-immunoprecipitation (Co-IP) studies, where YOD1 was tagged with Myc and TRIM54 was tagged with Flag and cotransfected into HEK 293T cells. When we immunoprecipitated the Myc-tagged proteins, it was clearly evident that the Flag-tagged protein also co-immunoprecipitated with Myc-tagged protein (Fig. 5*E*). Alternatively, when we immunoprecipitated Flag-tagged proteins, we could also observe the presence of Myc-tag protein (Fig. 5*F*). Since TRIM54 is an ubiquitinating enzyme, we wanted to assess if it binds and increases the ubiquitination of YOD1. Initially, we overexpressed TRIM54 and observed an increase in ubiquitination of endogenous YOD1 protein in HEK 293T cells (Fig. 6*A*). Next, we HA-tagged ubiquitin in cells overexpressing Myc-tagged YOD1 or Flag tagged TRIM54 and observed that overexpression of TRIM54 significantly increased HA-tagged ubiquitin levels (Fig. 6*B*). Finally, we performed Co-IP experiments in the presence of deubiquitinase 3. Initially, we observed that indeed ubiquitination of YOD1 increases in the presence of TRIM54, however presence of deubiquitinase 3 significantly decreased this ubiquitination (Fig. 6*C*). Hence, based on these results, it is evident that TRIM54 binds to and ubiquitinates YOD1 protein.

### TRIM54 decreases tendon injury in rat models

**Figure 7:**
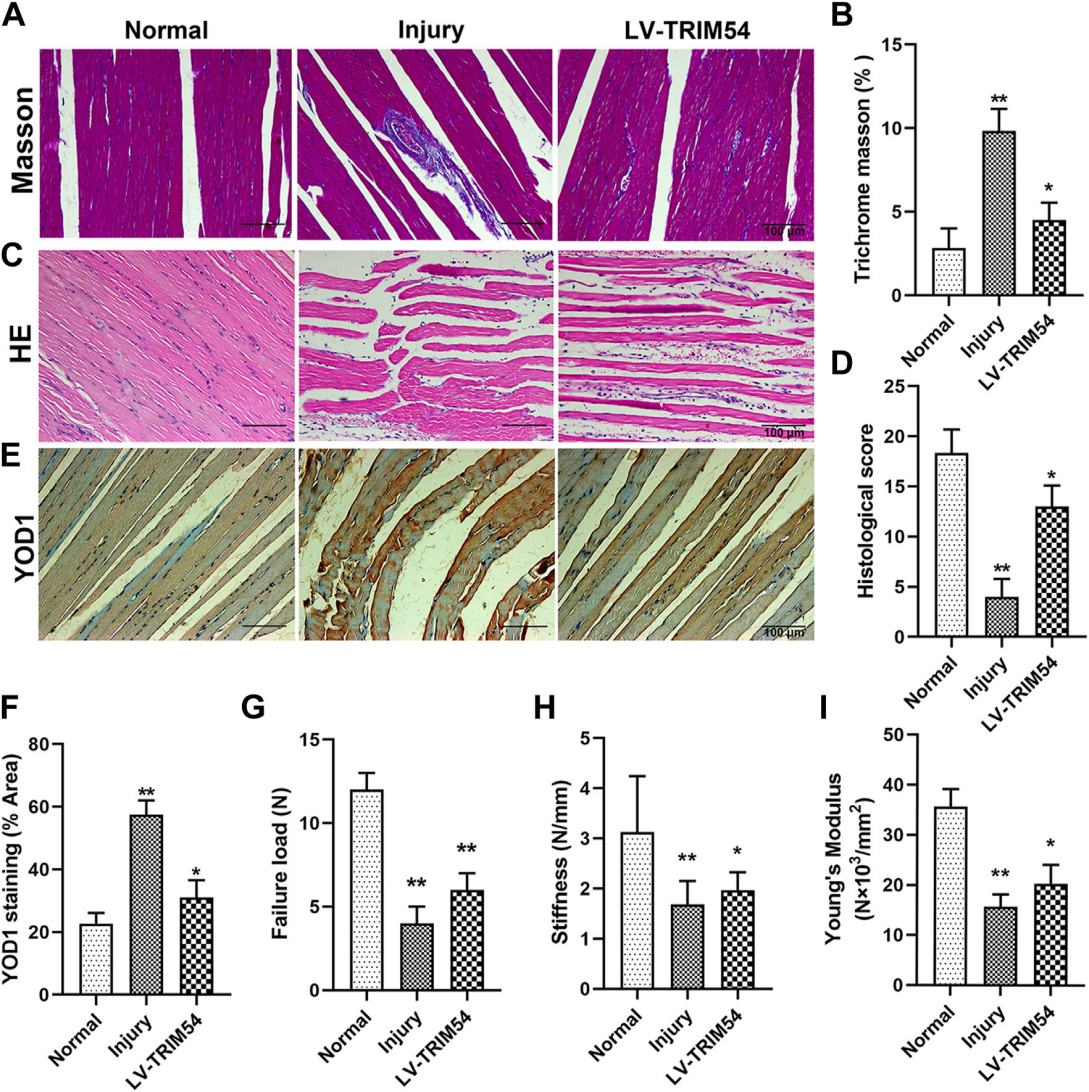
**TRIM54 decreases tendon injury in rat models.***A* and *B*, Masson’s staining was used to detect the effect of TRIM54 on the formation of collagen fibers in the process of tendinopathy in rat (Scale bar, 100 μm). *C* and *D*, HE staining was used to detect the effect of TRIM54 on the healing of Achilles tendon in rat and histological score of HE staining (Scale bar, 100 μm). *E* and *F*, immunohistochemical staining and quantitative analysis of YOD1 (Scale bar, 100 μm). *G*–*I*, tendon mechanical properties were assessed including failure load (*G*), stiffness (*H*), and young’ s modulus (*I*). n = 6 per group. Statistical significance was determined using one-way ANOVA and Tukey’s multiple comparisons test. Data are presented as mean ± SD. ∗*p* < 0.01, ∗∗*p* < 0.01 compared with the indicated groups. HE, hematoxylin–eosin; TRIM54, tripartite motif containing 54.

**Supporting Figure S3:**
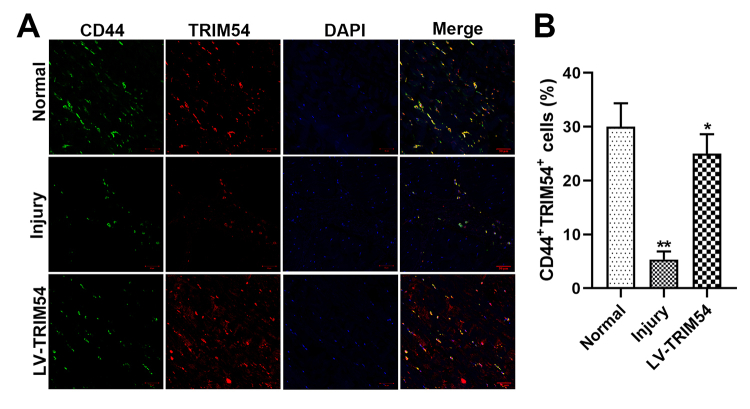
**Analysis of CD44**^**+**^**TRIM54**^**+**^**cells in rat Achilles tendon.***A*, immunofluorescence examination of Achilles tendon tissues from three groups of rat models reveals co-expression of CD44 (*green*) and TRIM54 (*red*) (Scale bar, 50 μm). *B*, bar plots showing number of CD44^+^TRIM54^+^ cells in Achilles tendon tissues from the three groups of rats. Statistical significance was determined using one-way ANOVA and Tukey’s multiple comparisons test. n = 6 per group. Data are presented as mean ± SD. ∗*p* < 0.01, ∗∗*p* < 0.01 compared with the indicated groups. TRIM54, tripartite motif containing 54.

We subsequently generated an *in vivo* tendon defect rat model and characterized the injury using hematoxylin–eosin (HE) staining and Masson’s trichrome staining. First, we examined the colocalization of mesenchymal stem cell markers CD44 and TRIM54 in Achilles tendon tissue of rats (Fig. S3, *A* and *B*). The findings suggested that the TRIM54 carrier had been transported in part to TDSCs. Indeed, we observed nonparallel, crumpled collagen fibers with cell accumulation in the injured rat tendons compared with the normal tendons which displayed parallel collagen and few embedded cells. Subsequently, we overexpressed TRIM54 in these injured rats and observed a rescue effect in morphology as observed through HE and Masson’s trichrome staining (Fig. 7, *A*–*D*). Similarly, immunohistochemical staining showed that the injury resulted in increased YOD1 staining compared with the control group, while overexpression of TRIM54 weakened the effect of the injury (Fig. 7, *E* and *F*). Additionally, we also assessed tendon mechanical properties and observed that injury significantly decreased failure load, stiffness, and young’s modulus. However, the rescued rats with TRIM54 overexpression showed significant improvement in all of the above mentioned mechanical properties (Fig. 7, *G*–*I*).

## Discussion

Presently, acute and chronic tendon injury are very common among both athletic and nonathletic populations (16). Symptomatically, the pain and the swelling associated with the injury are quickly addressed using nonsteroidal anti-inflammatory drugs (NSAIDs) such as corticosteroids (16). Further, rehabilitation using physical therapy gradually improves the functionality and use of the injured tendon. NSAIDs functions by inhibiting the production of pro-inflammatory prostaglandins. Importantly, NSAIDs exert both negative and positive effects (17). Increased prostaglandin during NSAIDs treatment can also aid in development of chronic tendinopathy. Hence, there is a strong need for alternative treatment strategies that aid in significant recovery after tendon injury.

Tendinopathy is understood to start with tendon degeneration followed by tearing, weakness, and pain (2). Hence, drugs focusing on regeneration along with decreasing inflammation are considered as important candidates. In this study, we initially observed that TRIM54 levels are significantly decreased in human tendinopathy samples compared to normal tendons. We further generated *in vitro* tendon inflammation model using TDSCs exposed to TNF-α. In these cells, we observed a significant decrease in TRIM54 expression levels and differentiation potential, along with increase in inflammatory markers and apoptosis. Interestingly, overexpression of TRIM54 significantly rescued these cells and improved TDSCs proliferation and differentiation (Figure 1, Figure 2, Figure 3, Figure 4). To further access the molecular pathway through which TRIM54 regulates the rescue of TDSCs, we performed downstream assays. Co-IP studies confirmed that TRIM54 binds and regulates YOD1 ubiquitination (Fig. 5). YOD1 is a highly conserved deubiquitinating enzyme of ovarian tumor family (18). Studies have indicated that YOD1 is a positive regulator of ITCH which then activates downstream YAP/TAZ thereby increasing the proliferation of liver hepatocytes (19). Additionally, studies have indicated YOD1’s role of deubiquitinating proteins in response to inflammatory cytokines such as IL-1 and subsequently antagonizes IKK/NF-kB signaling (18). In this study, we observed TRIM54 binds and increases ubiquitination of YOD1 (Fig. 6).

We also used *in vivo* models to further understand TRIM54’s role in tendon injury and observed that TRIM54 overexpression significantly improved the histopathology and mechanical properties of tendon injured animal model (Fig. 7). The current study illustrates the importance of understanding complex molecular mechanisms behind tendon injury. Indeed, understanding of tendinopathy and its etiology has evolved over years; however, the treatment options have been limited to just address the inflammatory clause of the disease. However, targeting candidates, such as TRIM54, could provide valuable alternative treatment options for treatment of tendinopathy.

## Experimental procedures

### Collection of human specimens

From March 2021 to October 2022, normal tendon tissue and varying degrees of tendinopathy were obtained from Shanghai Sixth People's Hospital. Twelve symptomatic patients with Achilles tendon disease (grade I-III, 8 males and 4 females, aged 38–70 years). The presence of Achilles tendinopathy was identified by ultrasound. All patients completed the VISA-A scoring system to evaluate the severity of Achilles tendinopathy. Eight patients who underwent anterior cruciate ligament reconstruction surgery had normal tendons (6 males and 2 females, aged 20–60 years) as controls. The tissue samples were then prepared for staining by fixing in formalin and embedding in paraffin. This study was approved by the Internal Review Board of the Institutional Ethics Committee of Shanghai Jiao Tong University Affiliated Sixth People's Hospital and abided by the Declaration of Helsinki principles. Informed consent was obtained from all participants prior to their participation in the study.

### Isolation and culture of TDSCs

TDSCs were isolated based on a previously published protocol (20). Briefly, TDSCs were obtained from male Sprague-Dawley rats. The Achilles tendons were separated from both limbs of each rat. Tendon tissues from all individuals were mixed and gently minced and digested with type I collagenase (3 mg/ml; Sigma-Aldrich) while passing them through a 70 μm cell strainer (Becton Dickinson) to yield a single-cell suspension. The cells were cultured in Dulbecco's modified Eagle's medium with 10% FBS, 100 U/ml penicillin, and 100 mg/ml streptomycin (all from Gibco), at 37 °C with 5% CO_2_. All experiments were performed with cells at passages 2 to 6, and the medium was changed every 3 days.

### Cell transfection

The coding sequence of TRIM54 overexpressed lentiviral vector was subcloned into pLVX-Puro lentiviral vector to construct its overexpressed lentiviral vector (LV-TRIM54). 293T cells were transfected with pLVX-Puro-TRIM54 using Lipofectamine 3000 (ThermoFisher Scientific). After 48 h, the recombinant lentiviral vector was purified and used to infect TDSCs. Cells transfected with empty pLVX-Puro were used as negative control (vector). Six hours after transfection, fresh medium containing 10% fetal bovine serum was replaced to maintain normal cell growth. qRT-PCR and Western blot were used to detect the transfection efficiency.

### TUNEL staining

To perform TUNEL staining, tissue samples were first fixed in 4% paraformaldehyde and permeabilized with 0.1% Triton X-100 in PBS. The samples were then equilibrated in equilibration buffer and incubated with the TUNEL reaction mixture containing TdT enzyme, dUTP, and reaction buffer. After incubation, the reaction was stopped by washing the samples in PBS and counterstaining the nuclei with a DNA-specific dye such as 4′,6-diamidino-2-phenylindole or propidium iodide. The samples are then observed under fluorescence microscopy or flow cytometry.

### Flow cytometry assay

Phosphatidylserine exposure on the surface of plasma membranes was detected using an Annexin V-FITC Apoptosis Detection Kit (Vazyme Biotech Co, Ltd) according to the manufacturer’s protocols. Briefly, cells were harvested with EDTA-free pancreatin, washed twice with ice-cold PBS, and resuspended in 100 μl of binding buffer. The cells were then incubated with 5 μl of Annexin V-FITC solution and 5 μl of propidium iodide staining solution at room temperature for 10 min in the dark. Finally, another 400 μl of binding buffer was added, and the cells were immediately analyzed by bivariate flow cytometry using a FACScan-LSR flow cytometer equipped with Cell Quest software (BD Biosciences). At least 1 × 10^4^ cells per sample were acquired and analyzed.

### Immunofluorescence

Initially, cells were seeded on coverslips placed on 6-well plates and rinsed in PBS, fixed in 4% paraformaldehyde for 15 min, then washed three times with PBS, and permeabilized with 0.1% Triton X-100 in PBS for 15 min. Further, cells were blocked with 5% BSA for 30 min and then incubated with CD146 antibody (Abcam, ab210072) and SOX2 antibody (Abcam, ab92494), Oct4 antibody (Abcam, ab19857) overnight at 4 °C, followed by FITC-labeled goat anti-rabbit IgG secondary antibodies (1:50; Bioss) for 1 h at 37 °C. Nuclei were labeled with 4′,6-diamidino-2-phenylindole (Beyotime) for 5 min. Images were captured under an inverted fluorescence microscope.

### Sphere formation assay

Cells were seeded on 96-well plate with 1000 cells per well. Cells were further cultured in sphere culture medium containing Dulbecco's modified Eagle's medium/F12, 2% B27, 20 ng/ml EGF, 20 ng/ml b-FGF, 4 μg/ml heparin, and 0.4% BSA for 1 week. The sphere formations were imaged with the aid of light microscope.

### Tendon defect model

For partially incised tendon injury, the Achilles tendon of Sprague Dawley male 6- to 8-week-old rats was exposed by a lateral incision under general anesthesia. The groups of the animals were as follows: normal group (Normal), Injury group, and Injury +TRIM54 overexpression group (LV-TRIM54). In the Injury group, for each leg, a gap wound (2 mm in width) was created by use of micro-operating instruments. The wound was then irrigated, and skin was sutured. Adenoviruses containing the TRIM54 overexpression vector were injected into the model group rats *via* the tail vein to overexpress the TRIM54. After operation, rats were allowed free cage activity with unrestricted access to normal food and drink. Rats were sacrificed for histological evaluation at 1 week postoperatively (21). This study was approved by the Internal Review Board of the Institutional Ethics Committee of Shanghai Jiao Tong University Affiliated Sixth People's Hospital.

### Multipotent differentiation

With regard to the differentiation experiment, TDSCs were cultured in 6-well plates (50,000 cells/well). Osteogenic, adipogenic, and chondrogenic were induced by a corresponding differentiation medium. The osteogenic differentiation medium contained a growth medium supplemented with 10 nM dexamethasone (Sigma-Aldrich), 5 mM β-glycerophosphate (APEXBIO), and 0.05 mM l-ascorbic acid 2-phosphate (Sigma-Aldrich). The adipogenic differentiation medium consisted of a growth medium supplemented with 500 μM isobutyl-methylxanthine, 60 μM indomethacin (Sigma-Aldrich), 0.5 μM hydrocortisone, and 10 μM insulin (Sigma-Aldrich). The chondrogenic differentiation medium was a Gibco StemPro chondrogenic differentiation kit (Thermo Fisher Scientific). The tenogenic differentiation medium contained a growth medium supplemented with 10 ng/ml TGF-β1 (Peprotech), 10 ng/ml GDF-5 (R&D System), and 0.05 mM l-ascorbic acid 2-phosphate. After a 2 week induction, Alizarin Red S, Oil Red O, and Alcian Blue were applied to evaluate osteogenic, adipogenic, tendon stem cell differentiation capacity, respectively (21).

### HE staining

After slides were dewaxed and hydrated using a dewaxing solution and gradient ethanol solution, they were soaked in hematoxylin for 3 min, then subjected to 5 s differentiation with 1% hydrochloric acid ethanol to turn the nucleus blue in color, and then lastly soaked in eosin for 2 min to stain the cytoplasm red. After dehydration with ethanol and clearing in a dewaxing solution, the slides were mounted. Images were acquired using an Olympus DP72 digital imaging system (Olympus Corporation).

### Masson staining

After dewaxing and hydration, slides were sequentially soaked in ponceau magenta for 10 min, 0.2% glacial acetic acid for 1 min, phosphomolybdic acid for 1 min, and 0.2% glacial acetic acid for 1 min to turn the cytoplasm red. Moreover, slides were treated with aniline blue for 30 s and soaked with 0.2% glacial acetic acid for 1 min to stain the fibrous tissue blue. The slides were subsequently dehydrated using ethanol, cleared in a dewaxing solution, and mounted. After image acquisition, the collagen volume fraction (ratio of blue dye area to red dye area) was calculated using Image-Pro Plus.

### Real-time reverse-transcription polymerase chain reaction

Total RNA was isolated from the primary cells and tendon tissues using a Trizol reagent (Thermo Fisher Scientific) according to the manufacturer’s instruction. Then, the mRNA was converted to complementary DNA by reverse transcriptase. The relative expression was calculated with the equation 2^–△△CT^. All reactions were performed in triplicate. The primers were given in the Table 1.

**Table 1:** Primer sequences for qRT-PCR

Genes	Species	Forward primer (5′-3′)	Reverse primer (5′-3′)
TNMD	Rat	GGACTTTGAGGAGGATGG	CGCTTGCTTGTCTGGTGC
COL1	Rat	CGAGTATGGAAGCGAAGG	AGTGATAGGTGATGTTCTGG
COL3	Rat	CTCCCAGAACATTACATACCA	GTCTTGCTCCATTCACCAG
SCX	Rat	AGAACACCCAGCCCAAACA	GTGGACCCTCCTCCTTCTAAC
Mkx	Rat	CAAACTAGGCTCTGGGTGGG	GTCAGTGTGTGCGGTGAAAC
IL-1β	Rat	TCCTCTGTGACTCGTGGGAT	TCAGACAGCACGAGGCATTT
IL-6	Rat	CCAGTTGCCTTCTTGGGACT	ACTGGTCTGTTGTGGGTGGT
iNOS	Rat	GTTCTCAGCCCAACAATACAAGA	GTGGACGGGTCGATGTCAC
TRIM11	Rat	ATCTTCCCTGAGACCCCCTT	AGAAGGTTTGGGTCACTGGG
	Human	CGAGACTCTGGCATGCTAAC	GTCCTCAGCTCCATAGGCAC
TRIM16	Rat	TGTTCACCCTTTGGCTCCTC	GCCCCAAGACAGAAGTCACA
	Human	TCGGTGTCAGAGGTCAAAGC	TCCATCTCGGCACTCCTGTA
TRIM21	Rat	CCTGGTAAGATTCCACGGCA	TCCCCCAACCTCAGAAATGC
	Human	AAACCCTGCCTCTTTTCCCC	CCATCTGGGGCAGGAATGTT
TRIM27	Rat	GGTTCCCGACACCCCTCT	CGCTCTCTCTCGGCTTCTC
	Human	TCCAGCATGTCACCCAGAAC	CAACCCTGGTCTACGCAGTT
TRIM54	Rat	CGAGGTGACTTTGGACGGG	GGGTAGCGCGATGGTCTCT
	Human	AGGAGGTGTGCCAGACTATC	GGTCGCCATACTGACGGATG
TRIM55	Rat	TGGGAAGCAACGACAGAGTC	TTCCTGTGTTCGGGTGATGG
	Human	GAGTCCACCAGGCCAGAAAA	GAGAGCAGGTGGGTACTTCG
TRIM63	Rat	GGGGAAGGAAGCCAAGTTCA	CCAATACCCAGCCCCTTCTG
	Human	GTCCAGCAGACACTGAACCA	CACCCAACGACCAGGCATTA
TRIM71	Rat	GGAGGGCTACATCATCGTGG	GATTGTCTTTGTCGGCCACC
	Human	CTCAGACACGAGGAACCACC	TGGCAGTCGGGGTGAATAAC
GAPDH	Rat	GATGGTGAAGGTCGGTGTGA	TGAACTTGCCGTGGGTAGAG
	Human	GAAAGCCTGCCGGTGACTAA	GCATCACCCGGAGGAGAAAT

### Western blot

Cell total proteins were extracted by Protein Extraction Kit (Beyotime) according to the manufacturer’s instructions and quantified by a bicinchoninic acid assay kit (Beyotime). In brief, after the cells were washed with PBS, the lysates were added and incubated on ice for 15 min, then were centrifuged at 4 °C. The proteins (20 μg per lane) were separated by 10% SDS-PAGE and were then transferred to PVDF membranes. Membranes were blocked with 5% nonfat milk in PBS containing Tween-20 at room temperature for 1 h and subsequently incubated with the following primary antibodies against: TRIM54 (Santa, sc-166137, 1:500); Sox2 (Abcam, ab92494, 1:1000); Tnmd (Abcam, ab203676, 1:1000); Oct4 (Abcam, ab18976, 1:500); COL1 (Santa, sc-59772, 1:1000); cleaved-caspase 3 (CST, #9661, 1:1000); and Bax (CST, #2772, 1:1000); YOD1 (Thermo Fisher Scientific, PA5-50157, 1:1000). After incubation at 4 °C overnight, membranes were three times washed with TBST, and then incubated with horseradish peroxidase–labeled secondary antibody for 1 h at room temperature. Finally, enhanced chemiluminescence kit (Pierce) was used to visualize protein bands, which were quantified using Image J software.

### Co-immunoprecipitation

Total protein samples were collected and lysed with RIPA lysis buffer (Beyotime, China, #P0013D) with protease inhibitor cocktail (Bimake, United States, #B14001), and the supernatants were collected by centrifugation at 12,000 rpm in a centrifuge. Each supernatant was mixed with primary antibodies against TRIM54 (Santa, sc-166137, 1: 100), YOD1 (Thermo Fisher Scientific, PA5-50157, 1: 50), and IgG at 4 °C overnight for endogenous IP. The next day, the lysates were incubated with 50 μl of protein ACG beads (Beyotime, China, #P2012) for 6 h at 4 °C. Furthermore, the beads were washed with RIPA three times to remove impurities. Finally, 20 μl of IP lysates was added to 2× loading buffer and boiled together. The results were analyzed by Western blots.

For Flag and Myc co-IP experiments, Flag-TRIM54 or Myc-YOD1 generated by Gateway technique (Invitrogen) was transfected into HEK 293T cells using Lipofectamine 3000. After transfection for 24 h, total protein samples were collected and lysed with RIPA lysis buffer with protease inhibitor cocktail, and the supernatants were collected by centrifugation at 12,000 rpm in a centrifuge. Anti-Myc (CST, #2276, 1: 200) and anti-FLAG (CST, #14793, 1: 50) were added and incubated overnight with each supernatant at 4 °C. Furthermore, the beads were washed with RIPA three times to remove impurities. Finally, 20 μl of IP lysates was added to 2× loading buffer and boiled together. The results were analyzed by Western blots.

### Immunohistochemistry analysis

The sections of Achilles tendons were washed in PBS, fixed in buffered formalin and 100% ethanol, embedded in paraffin, cut longitudinally to 5 μm thick sections, and mounted on 3-aminopropyl-triethoxy-silane–coated slides (Sigma-Aldrich). After deparaffinization, the sections were incubated with primary antibodies to TRIM54 (Thermo Fisher Scientific, MA5-26633, 1:150), CD68 (Abcam, ab955, 1:200), Col2 (Abcam, ab34712, 1:100), MMP13 (Abcam, ab3208, 1:100), c-caspase-3 ((CST, #9661, 1:400), and YOD1(Thermo Fisher Scientific, PA5-50157, 1: 100) overnight at 4 °C. After rinsing with PBS, an undiluted horseradish peroxidase–conjugated secondary antibody for 30 min at 37 °C. The slides were washed and incubated with 3,3′-diaminobenzidine chromogen substrate (Sigma-Aldrich). The nucleus was stained by hematoxylin (Sigma-Aldrich). Finally, cover the tissue sections with a glass coverslip and assess by light microscope.

### Statistical analysis

All statistical analyses were performed using GraphPad Prism Software, version 8.0 and SPSS software, version 19.0 (IBM). Differences between two or more groups were tested by unpaired Student’s *t* test or by one-way ANOVA with Tukey’s multiple comparisons test. A *p*-value less than 0.05 was considered statistically significance.

## Data availability

The datasets during the current study are available from the corresponding author on reasonable request.

## Supporting information

This article contains supporting information.

## Conflict of interest

The authors declare no conflict of interest with the contents of this article.
